# Comparison of evoked vs. spontaneous tics in a patient with trigeminal neuralgia (tic doloureux)

**DOI:** 10.1186/1744-8069-3-34

**Published:** 2007-11-06

**Authors:** David Borsook, Eric A Moulton, Gautam Pendse, Susie Morris, Sadie H Cole, Matthew Aiello-Lammens, Steven Scrivani, Lino R Becerra

**Affiliations:** 1 Pain/Analgesia Imaging Neuroscience (P.A.I.N.) Group, Brain Imaging Center, McLean Hospital, Harvard Medical School, Belmont, MA, 02478-1064, USA.; 2 Dept of Radiology, Athinoula A. Martinos Center for Biomedical Engineering, Harvard Medical School, Charlestown, MA, 02129-2020, USA.; 3 Cranio-Facial Program, New England Medical Center, Tufts University, Boston, MA, 02111-1817, USA.

## Abstract

A 53-year old woman with tic doloureaux, affecting her right maxillary division of the trigeminal nerve (V2), could elicit shooting pains by slightly tapping her teeth when off medication. The pains, which she normally rated as > 6/10 on a visual analog scale (VAS), were electric shock-like in nature. She had no other spontaneous or ongoing background pain affecting the region. Based on her ability to elicit these tics, functional magnetic resonance imaging (fMRI) was performed while she produced brief shocks every 2 minutes on cue (evoked pain) over a 20 min period. In addition, she had 1–2 spontaneous shocks manifested between these evoked pains over the course of functional image acquisition. Increased fMRI activation for both evoked and spontaneous tics was observed throughout cortical and subcortical structures commonly observed in experimental pain studies with healthy subjects; including the primary somatosensory cortex, insula, anterior cingulate, and thalamus. Spontaneous tics produced more decrease in signals in a number of regions including the posterior cingulate cortex and amygdala, suggesting that regions known to be involved in expectation/anticipation may have been activated for the evoked, but not spontaneous, tics. In this patient there were large increases in activation observed in the frontal regions, including the anterior cingulate cortex and the basal ganglia. Spontaneous tics showed increased activation in classic aversion circuitry that may contribute to increased levels of anxiety. We believe that this is the first report of functional imaging of brain changes in tic-doloureaux.

## Background

Trigeminal neuralgia, the most common craniofacial neuropathic pain disorder, is characterized by spontaneous, episodic, unilateral, electric-like shocks that arise from a consistent location in the face [1,2]. Of the three divisions of the trigeminal nerve, the second (V2) is most commonly affected. The pain of tic doloureaux can be excruciating and debilitating. Although a number of theories exist for trigeminal neuralgia, its mechanism remains unclear. Trigeminal neuralgia can arise spontaneously without apparent damage to the trigeminal nerve, but can also arise from compression or irritation of the dorsal root entry zone [3], or from damage such as tooth extraction [4]. Subjects with the condition have evoked pains (e.g., from touch, chewing, etc.) and/or spontaneous pain that emanates from the same location. The electric quality of this type of neuropathic pain differentiates it from the spontaneous burning pain or the evoked pains of allodynia and hyperalgesia, that are characteristic of other neuropathic pains [5].

Though several studies have evaluated electric stimulation in healthy subjects [6,7] and spontaneous and evoked pain activations have been compared in chronic pain patients [8-10], no study has yet evaluated the brain response in patients with pathological pain that was shooting/electric in nature. The aims of the study were (a) to determine a functional MRI (fMRI) paradigm that would allow us to measure pain associated with tics since a number of issues complicate this type of study including timing of the tic, movement associated with the tic (both facial spasm and head movement); (b) to describe brain activation associated with this type of pain; (c) to differentiate between 'evoked' pain produced by cues and spontaneous pain in this patient and (d) to determine if there were any differences observed for activation patterns by tic pain with other pain activations in chronic pain patients.

## Case presentation and methods

The investigation was approved by the McLean Hospital IRB Ethics Committee. The study met with the ethical standards as defined by the Helsinki agreement on human experimentation. The subject was compensated for her participation.

## Patient history

The patient is a 53-year-old female diagnosed with trigeminal neuralgia in 2002. Prior to the onset of her pain, she had extensive dental work (two root canals and three extractions). No other contributing factors were present in her history. The pain was sharp, electrical in nature, and primarily affected the right V2 region, specifically the upper lip. Her pain was a recurring, temporally discrete experience that lasted for a few seconds (< 1–2 seconds) at a time and radiated laterally towards her ear. The pain attacks were sometimes accompanied by a mild motor tic. The patient experienced both evoked (e.g., by tapping her teeth, eating, brushing her teeth, movement of the jaw, wind against her face, touch) and spontaneous shooting pain that had no obvious precipitating factor. After discontinuation of her medication for a day, the patient could evoke these shock pains by light tapping of her teeth. Typically, the patient had no background pain between attacks unless she experienced multiple tic attacks over a short period of time. The patient rated her worst tic-related pain as 8–10/10.

Her usual medications included carbamazepine (Tegretol, 300 mg/day) and gabapentin (Neurontin, 900 mg/day); these provided excellent pain relief. If she discontinued the medications for 12 hours she could evoke tics (see below) by tapping her teeth lightly. She had no other significant medical history.

Prior to scanning, the patient underwent a battery of testing including forms to evaluate depression (Beck Depression Scale – BDI-II) and the Galer/Jensen Neuropathy Pain Scale (NPS) and McGill Pain Questionnaire (MPQ). The patient scored a 5/63 on the BDI-II, indicating that she was not depressed. The scores for the NPS, each rated on an 11-point scale for pain quality were: intense 8, sharp 8, hot 1, dull 1, cold 1, sensitive 1; itchy 1, pain with standing or walking 5, unpleasant 8, deep pain rating 2, and surface pain rating 8. On the MPQ the patient scored a 20/78 on the PRI (pain rating index) section of the form. She scored a 4/5 on the PPI (present pain intensity) section of the questionnaire. The patient indicated that the following words best described her pain: pulsing, flashing, lancinating, sharp, exhausting, intense, piercing, and horrible.

## Functional magnetic resonance imaging (fMRI)

MRI was carried out in a 3.0 T Siemens Trio scanner (Erlangen, Germany) with a quadrature head coil. For anatomical localization, an MPRAGE was used (1 × 1 mm in-plane resolution, 1.3 mm slice thickness). Magnitude and phase images were acquired on the same orientation as the functional scan to correct for susceptibility distortions. Functional scans were acquired using a Gradient Echo (GE) EPI sequence with isotropic resolution of 3.5 mm, 41 slices (no-gaps) were prescribed obliquely along the brainstem axis. A TR/TE = 3.0 s/30 ms was used and 404 volumes were acquired.

**Figure 1:**
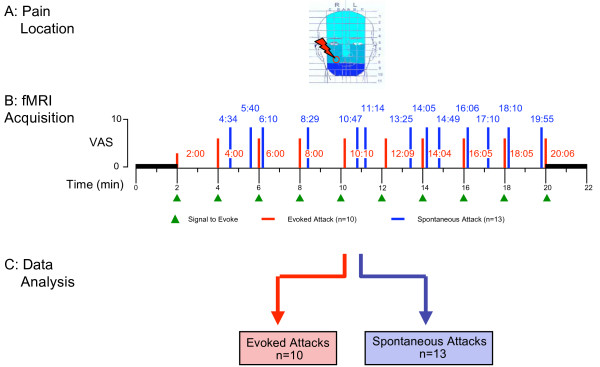
fMRI paradigm

In order to determine brain areas activated by the pain, fMRI was used to measure regional hemodynamic changes related to the timing of her shock-like pain. Following a short practice session, she underwent standard anatomical scans followed by a 24-minute functional scan (Figure 1). During the functional scan, she would tap her teeth once or twice in response to a light tap to the maleolus of the left foot. After a baseline scan of 2 minutes, the tap cue was administered every 2 minutes, followed by a 2-minute baseline scan at the end of the session. The timing of each tic attack was determined by the patient signaling its onset by turning a dial using the hand contralateral to the side of the tic. Since the duration of the tics were consistent and very short (< 2 × the image repetition time (TR = 2.5 sec)), offset was not marked. The times for each tic (evoked and spontaneous) are noted in Figure 1. The subjects rated pain intensity for evoked and spontaneous pains on a computerized VAS (0–10) upon completion of the scan.

Activation in the ganglion and trigeminal nucleus could not be assessed as we have previously done [9,11] as these areas were severely affected by signal artifacts from metal dental work.

## Data analysis

### General Analysis

Analysis was carried out using FEAT (FMRI Expert Analysis Tool) Version 5.43, part of FSL (FMRIB's Software Library [68]). The following pre-statistics processing was applied; high-pass filtering for trend removal and spatial smoothing FWHM = 10 mm to improve signal to noise ratio. There were two binary (0 = on, 1 = off) explanatory variables (EVs). The EV's were convoluted with standard hemodynamic responses (fsl). The first EV modeled the evoked pain, and the second EV modeled the spontaneous pain. General linear model (GLM) based time-series statistical analysis was carried out using FILM (FMRIB's Improved Linear Model) with local autocorrelation correction [12]. Statistical maps corresponding to the evoked, spontaneous, and evoked vs. spontaneous were created and thresholded using Gaussian Mixture Modeling (GMM) with automatic model order selection using Bayes Information Criterion. GMM is a multiple comparisons-based analysis generally used for unsupervised classification of data into multiple categories [13], and was used to determine appropriate z-statistical thresholds. No additional criteria using spatial extent were used to determine significance.

### Evaluation for Motion

To ensure that responses to evoked and spontaneous EVs were not contaminated by motion, we tested for a significant correlation between the EVs and measures of head motion. A general linear model (GLM) analysis was run between the design matrix and the three translations along x, y, and z directions estimated during motion correction. An F-test for overall model fit was used to determine any significant correlation between the design matrix and the motion parameters.

### Single Trial Average

In order to verify that changes in the BOLD signal correlated with the spontaneous or evoked tic, single trial averages (STA) were evaluated. STA were calculated using in-house programs implemented via MATLAB (Release 7.2, Mathworks Inc., Natick, MA, USA) in combination with the functional time course and stimuli activation maps. Two regions of interest were defined to encompass the left and right insula in functional resolution space. The insula was chosen as a representative region since it is involved in both sensory [14] and emotional reactions to pain [15]. Furthermore, the insula is not expected to participate in the motor network that may be minimally utilized during the patient's marking of tic events. Direct stimulation of the insula in humans can produce intense shock-like pain [16], and this area is one of the most consistently activated brain regions during pain [17,18]. Other regions significantly activated would be expected to also show responses, although the temporal profile of their responses may be different. Activation masks for the evoked and spontaneous tics for both ROIs were created based on GMM-determined thresholding of the z-statistics (z > 5.93 for increased activation and z < 2.96 for decreased activation for evoked stimulus; z > 2.24 for increased activation and z > 0.85 for decreased activation for spontaneous stimulus; z > 2.86 for evoked > spontaneous and z < 1.98 for spontaneous > evoked). The mean time course for each ROI was extracted from the high pass-filtered and spatially smoothed functional image. The EV for each stimulus type (evoked and spontaneous) was sampled to define each specific "trial". A trial was defined as the period consisting of 3 seconds prior to the beginning of the tic attack (as indicated by the patient's use of the dial), and 33 seconds immediately following the onset of the tic attack. A trial average was calculated for each ROI and each stimulus by taking the average time course of the trials. To avoid the possibility of calculating temporally overlapping tic-responses, only tics that were spaced at least 36 seconds apart were considered (evoked n = 3; spontaneous n = 3). This criterion was determined during preliminary analysis, which indicated that responses of isolated tics returned to baseline within this time frame. For the statistical analysis, however, all the evoked and spontaneous tics were included.

## Results

### Pain intensity score within scans

Evoked vs. Spontaneous Pain: The patient rated her pain immediately after the scanning sessions. For evoked pain, the first event was rated 5/10 and subsequent evoked episodes were rated a 6–7/10. For spontaneous pain, she rated all episodes as 8/10. The cumulative repetitive shocks did not seem to induce any exacerbation of her pain scores over time, at least as assessed by the stable nature of her pain scores. Note, however, that these pain ratings were recorded immediately after the end of functional scan acquisitions.

### Internal controls

**Figure 2:**
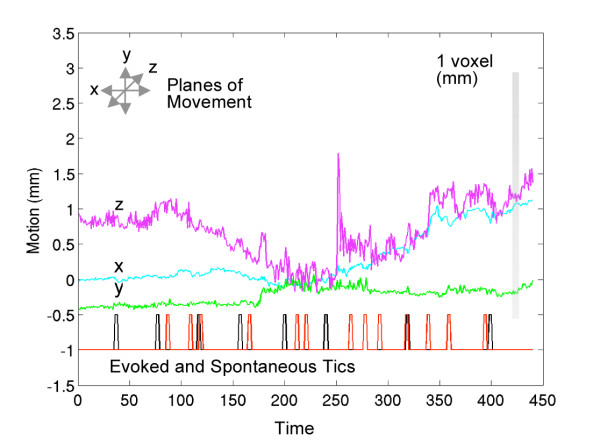
**Evaluation of motion during fMRI acquisition**. Estimated translations along x (right-left), y (anterior-posterior), and z (superior-inferior) directions during motion correction. The bar on the right shows the voxel size relative to these translations. The evoked (black) and spontaneous (red) EVs used in the GLM-based analysis of functional data are displayed at the bottom of the graph. An F-test investigating the correlation between motion parameters (x, y, and z) and the design matrix used in the analysis indicates no significant correlation.

Test for head movement: Figure 2 shows motion in the right-left (x), anterior-posterior (y) and superior-inferior (z) domains. For a GLM on the individual translations with the full design matrix, an F-test for overall model fit gives the following p-values: for x: p = 0.1031; y: p = 0.9453; and z: p = 0.8142. Since none of these p-values are significant, we rule out any significant correlation between the design matrix and the motion parameters, thereby eliminating head motion as a potential confound in the generation of the evoked and spontaneous activation maps.

### CNS activation by evoked tics

**Figure 3:**
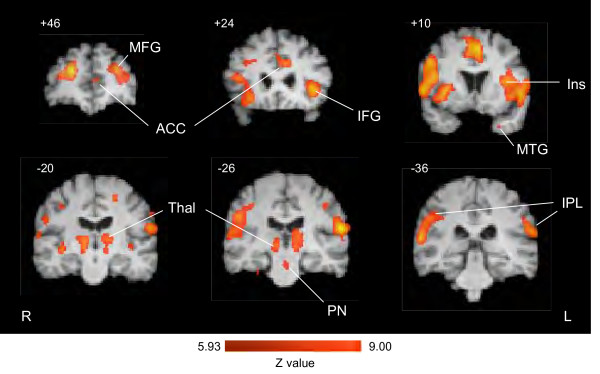
**Activation by evoked tics**. Activation maps based on GLM-based analysis using the evoked EV (n = 10 tics). A number of cortical regions including anterior cingulate (ACC), insula (Ins), middle and inferior frontal (MFG, IFG), medial temporal gyrus (MTG) and inferior parietal lobe (IPL) regions show significant activation (P < 0.0001). Subcortical regions showing significant activation include the thalamus (Thal) and pontine nuclei (PN). Notably, no significant activation was observed in the cerebellum (see text). Numbers indicated the anterior posterior, sagittal or horizontal plane of the brain slice. R = Right and L = Left.

Activation maps were defined on the basis of the 10 evoked tics are shown in Table 1 and 2 and examples are shown in Figure 3. Significant increased activation (z-value > 5.93) was found in a number of cortical regions (the frontal, parietal, and temporal cortices, cingulate, and insula cortices), as well as sub-cortical regions (thalamus, basal ganglia and pontine nuclei). The total number of activations above threshold was 33. A number of foci were significantly activated in the superior, middle, and inferior frontal cortex (see Table 1). Activation in the cingulate was bilateral and included the genual, mid ACC, and postgenual ACC. Focal activation was seen in the mouth representation of S1 (postcentral gyrus [PoCG] BA3 and BA2). Activation in the M1 region was observed in 2 foci, one ipsilateral to the pain and one contralateral to the hand used for indicating a tic-event (see above). Significant bilateral activation was seen in the thalamus and in the claustrum. The brainstem was notable for increased activation in pontine nuclei (PN). Decreased activation was observed in the hypothalamus. Of note, no activation was observed in the cerebellum, a region that is active in many pain studies.

**Table 1:** Evoked tics (increased signal)

	Brain Region*	Zmax	Vol. (cc)	MNI x	MNI y	MNI z
**Cortical Regions**						
*Frontal Lobe*						
	SFG (10)	8.10 (I)	3.33	24	47	20
		7.11 (C)	1.92	-25	43	26
	SFG (6)	7.28 (I)	2.83	17	-3	60
	MFG(6)	8.02 (C)	3.65	-26	-12	56
	IFG (13)	6.77 (I)	1.66	46	26	11
	IFG (46)	6.86 (C)	2.56	-44	39	14
	IFG (47)	7.75 (C)	4.51	-45	16	-6
	IFG (9)	7.56 (I)	2.31	52	9	32
	TTG (42)	6.33 (I)	0.26	66	-17	12
*Motor Cortex*						
	PreCG (6)	7.62 (C)	3.60	-56	3	13
		7.61 (I)	1.92	43	-1	42
*Sensory Cortex*						
	PoCG (3)	6.33 (I)	0.30	39	-24	43
	PoCG (2)	6.97 (I)	3.42	54	-28	32
*Temporal Lobe*						
	STG (22)	7.24 (I)	2.99	46	14	54
		7.75 (I)	3.14	59	9	1
	MTG (39)	6.26 (C)	0.30	-53	-59	10
*Parietal Lobe*						
	IPL (40)	8.29 (C)	4.84	-61	-24	18
*Cingulate Cortex*						
	ACC (24)	7.31 (C)	1.50	-9	20	28
	ACC (32)	6.96 (C)	0.84	-16	37	24
		7.88 (B)	7.64	-1	10	49
*Insula*						
	Ins (13)	7.54 (I)	5.42	35	15	0
		7.44 (I)	1.67	47	10	17
		7.32 (C)	3.62	-37	9	12
		7.12 (I)	1.78	39	2	-4
		7.05 (C)	0.53	-29	5	-39
		7.34 (C)	0.87	-40	3	23
		6.36 (C)	0.62	-40	-13	-6
**Subcortical Regions**						
*Thalamus*						
	Thalamus	6.95 (I)	2.12	14	-16	6
		6.44 (C)	0.67	-11	-26	-2
	Pulvinar	6.72 (C)	1.38	-12	-24	7
*Basal Ganglia*						
	Claustrum	7.17 (C)	2.56	28	24	32
		6.78 (I)	0.95	38	-19	4

ACC – anterior cingulate cortex; IFG – inferior frontal gyrus; Ins – insula; ITG – inferior temporal gyrus; MFG – middle frontal gyrus; MTG – medial temporal gyrus; PN – pontine nuclei; PoCG – postcentral gyrus; PreCG – precentral gyrus; SFG – superior frontal gyrus; STG – superior temporal gyrus; TTG – transverse temporal gyrus.

*Brain Region – name and where appropriate Brodmann Area ( ). MNI – Montreal Neurological Institute

**Table 2:** Evoked tics (decreased signal)

	Brain Region*	Zmax	Vol. (cc)	MNI x	MNI y	MNI z
**Cortical Regions**						
*Cingulate Cortex*						
	ACC	6.23 (C)	8.82	-9	32	0
**Subcortical Regions**						
*Hypothalamus*						
	Hyp	3.90 (B)	2.86	-3	-2	-13
**Brainstem and Cerebellum**						
*Medulla*						
	Medulla	3.90 (C)	1.04	-5	-48	-55

ACC – anterior cingulate cortex; Hyp – hypothalamus

*Brain Region – name and where appropriate Brodmann Area ( ). MNI – Montreal Neurological Institute

Table 2 shows that significantly decreased activation resulting from evoked tics (z value > 2.96) occurred in only a few regions (anterior cingulate cortex, hypothalamus, and the medulla).

### CNS activation by spontaneous tics

**Figure 4:**
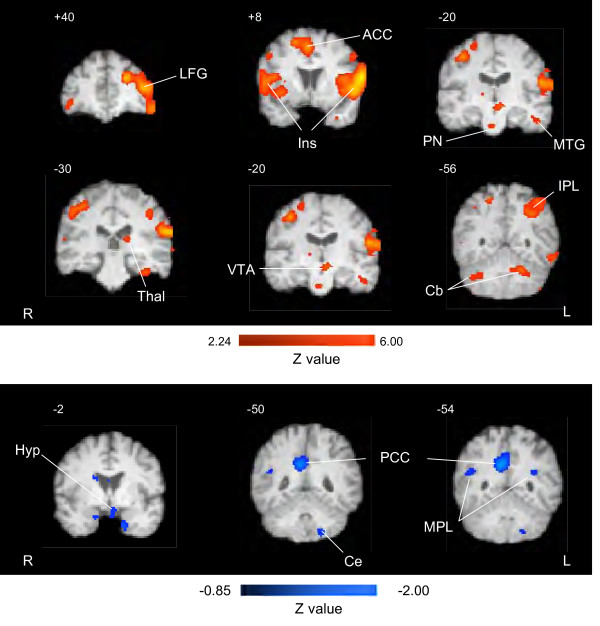
**Activation (increased top panel and decreased lower panel) in brain regions by spontaneous tics**. Activation maps based on GLM-based analysis using the spontaneous EV (n = 13 tics). Increases were observed in a number of areas (See Table 3) and examples of these are shown here. Activation was present in the lateral frontal gyrus (LFG), the anterior cingulate (ACC), Insula (Ins), posterior thalamus (Thal), pontine nuclei (PN), ventral tegmental area (VTA), cerebellum (Cb) and inferior parietal lobe (IPL). Spontaneous tics significantly decreased baseline levels of brain activity in several areas. These include the anterior hypothalamus (Hyp), posterior cingulate cortex (PCC), and middle parietal lobe (MPL). R = Right and L = Left.

Table 3 shows foci of increased activation that reached significance based on statistical level (z value ≥ 2.24). A total of 40 regions showed increase in activations that met the statistical threshold. Examples of activations are shown in the activation maps in Figure 4, top panel. Overall there was a similar distribution of activation to that observed for evoked tics; however there were more activations observed in the frontal, temporal and parietal lobes compared with the evoked tics. The only thalamic region activated was the pulvinar nucleus (posterior thalamus). Two regions that were activated, that were not observed in the evoked group, included the ventral tegmental area (VTA) and the cerebellum, with the latter showing bilateral activation.

**Table 3:** Spontaneous tics (increased signal)

	Brain Region*	Zmax	Vol. (cc)	MNI x	MNI y	MNI z
**Cortical Regions**						
*Frontal Lobe*						
	MFG (6)	3.25 (I)	1.31	19	8	56
		2.91 (C)	0.43	-38	2	47
		3.57 (B)	7.81	2	2	52
		3.39 (C)	2.48	17	-9	62
		2.96 (C)	0.46	-34	-9	52
	MFG (9)	3.84 (C)	4.15	-22	43	24
		2.81 (I)	0.54	52	5	41
	MFG (10)	3.20 (C)	1.59	-47	55	1
		2.60 (I)	0.43	42	51	-4
		4.36 (C)	7.79	-42	49	15
		3.35 (I)	2.67	38	-23	52
	MFG (46)	3.18 (C)	1.69	-38	30	25
	IFG (47)	2.65 (I)	0.44	49	41	-9
		3.53 (C)	3.83	-57	35	-14
		3.49 (I)	4.22	32	22	-5
	IFG (9)	2.96 (C)	0.81	-52	5	38
		2.41 (C)	0.29	-51	-17	-27
	IFG (44)	4.54 (C)	11.38	-63	9	12
		3.14 (I)	0.85	47	9	13
		3.24 (I)	3.96	56	8	9
*Motor Cortex*						
	PreCG (6)	2.60 (I)	0.46	26	-21	65
*Temporal Lobe*						
	STG (38)	2.76 (C)	1.04	-53	17	-23
	STG (22)	4.36 (C)	5.05	-53	11	-5
	MTG (21)	2.80 (C)	1.19	-37	-3	-31
	ITG (37)	2.83 (C)	0.97	-60	-61	-11
*Parietal Lobe*						
	IPL (40)	4.71 (C)	7.04	-61	-25	21
		3.23 (I)	3.58	45	-35	48
		3.62 (C)	1.52	-63	-43	32
		3.45 (C)	4.39	-52	-46	38
*Insula*						
	Ins (13)	4.21 (C)	6.03	-37	24	1
		2.57 (C)	0.77	-38	-9	-3
		2.82 (I)	0.53	37	-14	4
*Cingulate Cortex*						
	ACC (32)	3.43 (B)	6.15	1	27	38
		2.96 (I)	0.80	16	20	51
**Subcortical Regions**						
*Thalamus*						
	Pulvinar	2.72 (C)	0.38	-17	-32	12
**Brainstem and Cerebellum**						
*Ventral Tegmental Area*						
	VTA	2.88 (C)	0.73	-5	-20	-11
*Cerebellum*						
	Cb	3.23 (C)	2.45	-19	-60	-30
		2.45 (I)	0.48	33	-59	-39
		3.57 (C)	2.78	-26	-74	-56

ACC – anterior cingulate cortex; IFG – inferior frontal gyrus; Ins – insula; IPL – inferior parietal lobe; ITG – inferior temporal gyrus; MFG – medial frontal gyrus; MTG – medial temporal gyrus; PN – pontine nuclei; PreCG – precentral gyrus; STG – superior temporal gyrus; VTA – ventral tegmental area; Cb – cerebellum.

*Brain Region – name and where appropriate Brodmann Area ( ). MNI – Montreal Neurological Institute

Table 4 shows foci of decreased activations following spontaneous tics. A total of 8 foci reached statistical levels of activation (z ≥ 0.85). Examples of activations are shown in the activation maps in Figure 4 The regions, many considered to be involved in aversive responses to pain [19] activated included the posterior cingulate cortex, hypothalamus, amygdala, and hippocampus.

**Table 4:** Spontaneous tics (decreased)

	Brain Region*	Zmax	Vol. (cc)	MNI x	MNI y	MNI z
**Cortical Regions**						
*Motor Cortex*						
	PreCG (4)	1.41 (C)	1.02	-57	-10	39
*Temporal Lobe*						
	STG (22)	1.10 (I)	0.46	44	-53	22
	MTG (39)	1.26 (C)	0.33	-33	-56	22
*Cingulate Cortex*						
	PCC (31)	1.51 (C)	1.19	-13	-64	26
	PCC (23)	2.81 (B)	6.88	6	-55	31
**Subcortical Regions**						
*Hypothalamus*						
	Hyp	1.57 (C)	4.05	-4	5	13
*Amygdala*						
	Amy	1.20 (I)	0.50	18	-7	-24
*Hippocampus*						
	Hip	1.33 (C)	0.55	-28	-19	-15

Amy – amygdala; Hip – hippocampus; Hyp – hypothalamus; MTG – medial temporal gyrus; PCC – posterior cingulate cortex; PreCG – precentral gyrus; STG – superior temporal gyrus.

*Brain Region – name and where appropriate Brodmann Area ( ). MNI – Montreal Neurological Institute

### Evoked vs. spontaneous Tics

**Figure 5:**
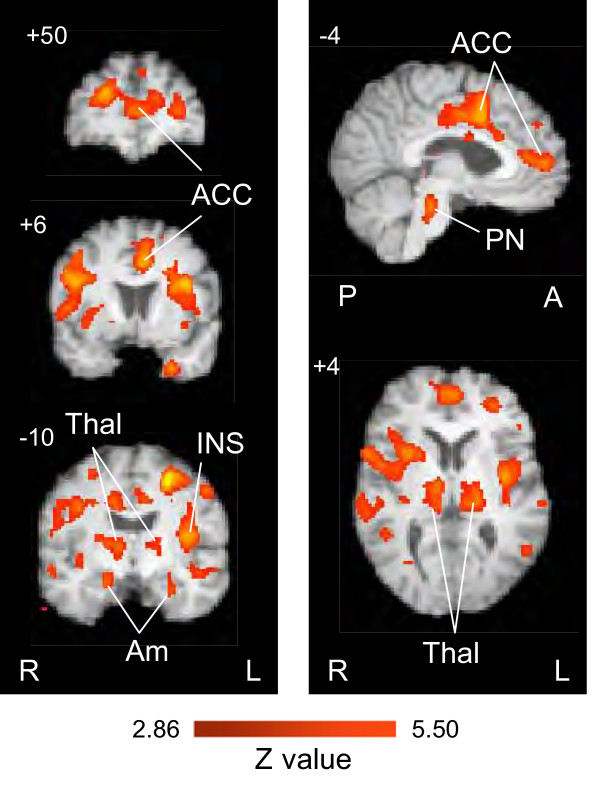
**Contrast map of evoked > spontaneous tics**. Contrast maps for Evoked > Spontaneous pain. See Table 3. Key: ACC – anterior cingulate cortex; Ins – Insula; Amy – amygdala; Thal – thalamus; PN – pontine nuclei. R – right; L – left; P – posterior; A – anterior.

Differences in activation between evoked and spontaneous tics might result from differences in expectancy or anticipation or the severity of the attack. We therefore performed contrast analyses for differences between these two conditions for evoked > spontaneous (Table 5; Figure 5) and for spontaneous > evoked (Table 6).

**Table 5:** Evoked tics > spontaneous tics

	Brain Region*	Zmax	Vol. (cc)	MNI x	MNI y	MNI z
**Cortical Regions**						
*Frontal Lobe*						
	SFG (10)	3.17 (I)	0.51	12	60	20
	MFG (10)	3.90 (I)	5.79	1	57	8
		3.37 (C)	1.04	-13	53	16
	MFG (9)	4.85 (I)	6.93	24	45	19
		4.10 (I)	4.77	36	27	29
	MFG (6)	3.44 (I)	1.94	28	0	55
		4.86 (C)	4.93	-26	-12	54
	IFG (9)	4.41 (I)	6.83	49	10	29
	IFG (47)	3.58 (I)	2.40	36	22	-19
		3.32 (C)	1.61	-42	18	-9
	IFG (13)	4.07 (I)	3.36	46	26	11
*Temporal Lobe*						
	STG (22)	3.71 (I)	1.74	59	11	1
		3.28 (C)	1.35	-51	-18	-10
		3.26 (I)	1.39	66	-23	7
		3.49 (C)	1.24	-63	-37	20
		3.49 (C)	1.45	-66	-38	13
		3.70 (C)	3.27	-53	-53	17
	STG (38)	3.87 (C)	0.98	-26	6	-39
	STG (39)	3.42 (I)	0.87	47	-52	12
	MTG (21)	3.53 (I)	1.97	58	-17	-15
		3.43 (I)	2.31	70	-32	-6
	MTG (20)	3.57 (I)	1.81	53	-33	-12
	MTG (37)	3.56 (I)	1.19	51	-48	-6
*Parietal Lobe*						
	IPL (40)	4.00 (I)	2.76	60	-32	17
		3.30 (C)	0.62	-55	-37	32
	Precun (7)	3.46 (C)	0.85	-21	-51	54
*Motor Cortex*						
	PreCG (6)	4.32 (I)	5.49	43	-3	41
		3.81 (C)	3.16	-42	-17	38
	PreCG (4)	3.94 (I)	2.12	39	-23	38
	PreCG (43)	3.27 (I)	0.84	62	-5	14
	SMA (6)	3.08 (C)	0.37	-16	9	60
*Sensory Cortex*						
	PoCG (2)	4.55 (I)	8.64	51	-25	28
	PoCG (3)	3.41 (C)	0.31	-25	-36	60
*Cingulate Cortex*						
	ACC (24)	3.80 (C)	1.72	-9	18	29
		4.29 (C)	6.46	-6	7	43
		3.66 (I)	3.81	13	-20	45
	ACC (32)	3.72 (C)	2.72	-16	37	24
		3.33 (C)	0.80	11	14	42
*Insula*						
	Ins (13)	4.57 (C)	5.99	-37	7	24
		4.24 (C)	7.37	-39	-7	11
**Subcortical Regions**						
*Basal Ganglia*						
	Claustrum	3.81 (I)	2.77	33	13	3
		3.79 (I)	4.01	39	-2	-3
		3.97 (I)	1.78	38	-19	-6
		3.53 (C)	1.63	-40	-23	-2
*Thalamus*						
	Thalamus (vl)	3.65 (I)	2.97	11	-15	5
		3.76 (C)	5.03	-20	-25	-1
		3.40 (C)	0.35	-9	-25	15
		4.08 (I)	5.13	12	-27	-5
**Brainstem and Cerebellum**						
*Pons*						
	PN	3.89 (I)	1.98	11	-29	-37
		3.61 (C)	1.39	-4	-28	-24

ACC – anterior cingulate cortex; IFG – inferior frontal gyrus; Ins – insula; IPL – inferior parietal lobe; MFG – medial frontal gyrus; MTG – medial temporal gyrus; PN – pontine nuclei; PoCG – postcentral gyrus; PreCG – precentral gyrus; SFG – superior frontal gyrus; STG – superior temporal gyrus; vl – Ventrolateral thalamus.

*Brain Region – name and where appropriate Brodmann Area ( ). MNI – Montreal Neurological Institute

**Table 6:** Spontaneous tics > evoked

	Brain Region*	Zmax	Vol. (cc)	MNI x	MNI y	MNI z
**Brainstem and Cerebellum**						
*Medulla*						
	Medulla	3.24 (C)	2.20	-2	-43	-54

For the contrast analysis of evoked > spontaneous, 50 regions achieved significance (z value > 2.86). The distribution was similar to that shown for activations by evoked alone, suggesting that the overall effect for evoked was greater than spontaneous tics. Though the evoked were generally greater than spontaneous tic-related activations, the pain reports for the latter were greater than for the evoked tics. A relatively large level of activation was also observed in the pontine reticular formation (Figure 5). In contrast to this, the number of regions that reached significance for decreased activation for the spontaneous tics was larger (n = 8) compared with the evoked tics (n = 3). For spontaneous > evoked, the contrast analysis showed that only 1 region, the brainstem, reached significance (z value ≥ 1.98). A similar parity for this contrast was observed for activation in various regions when comparing Tables 1 and 2, with 33 foci showing increased activation by the evoked tics and 40 by the spontaneous tics (see comparison below). Only sub-threshold differences were observed in the cerebellum.

#### Single trial average (STA)

**Figure 6:**
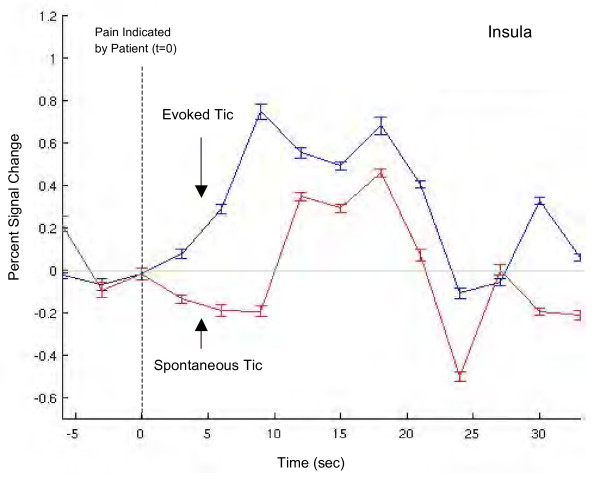
**Single trial average**. Single trial average BOLD responses of left and right insula during evoked and spontaneous stimuli (see text for details). Percent signal change was calculated based on the following: (y - $\bar{\text{y}}$ * 100)/$\bar{\text{y}}$, where y represents the mean time series and $\bar{\text{y}}$ is the mean of the mean time series. Error bars represent standard error of the mean across trials. Note the early onset of the BOLD response following the evoked tic (see text).

Figure 6 shows STA responses (mean ± SEM) for the evoked and spontaneous tics for the insula. Note that the onset of the BOLD response occurs earlier for the evoked tic vs. the spontaneous tic, and that the percent signal change is greater. Evoked and spontaneous single trial averages for the left and right insula show similar response patterns (data not shown). The evoked response STA shows a gradual increase until it reaches maximum value while the spontaneous STA lags in time before rapidly reaching its maximum value. Furthermore, it is possible to observe the two peak response to the pain that previously has been observed following acute pain in healthy volunteers [19]. If the responses in the insula were contaminated by the motor task used to mark tics, the response onset time for spontaneous and evoked tics would presumably be similar. As the onset of the evoked tic began ~3 seconds after the patient indicated pain, and the spontaneous tic response began ~12 seconds after, this was not the case. We interpret this as indicating that the influence of motor activity (hand motion) on the response in the insula was minimal.

## Discussion

Tic doloureaux is a severe and relatively common facial pain disorder that has an unusual presentation for a neuropathic pain condition [20,21]. The patient did not have any tics in the prior weeks because of successful pharmacological therapy (see above), but had induced some after coming off the drug prior to the scanning session. Here, slight tapping of the teeth "evoked pain" as a result of triggering the tic. The pain of a tic-produced activation in a number of regions associated with cognitive, sensory, and emotional functions.

### CNS activation common to evoked and spontaneous tics

A large portion (anterior, middle, and posterior regions) of the anterior cingulate cortex was active during both evoked and spontaneous tics. This is consistent with pain studies demonstrating activation in this region [18], though differing in the extent of the activation. The differences may relate to a number of issues including anticipation of pain, severity of the pain, the patient's familiarity with the pain, and the patient indicating pain during the scan with the dial.

Furthermore, differences in cingulate activation were observed between evoked and spontaneous tics in the posterior cingulate, where a significant decrease in activation was observed only for spontaneous tics. Posterior cingulate activation has been previously linked to pain by intracutaneous electrical stimulation [22], by allodynia in complex regional pain syndrome [10], by pain related fear and anxiety [23], and following central sensitization in healthy volunteers [24]. Previous reports have shown posterior cingulate involvement in monitoring and evaluation of affective responses [25]. It is also considered to be involved in a neural network of conscious awareness [26]. In this subject, the onset of the spontaneous tics was not predetermined, and the activation observed here may reflect information processing during aversive sensation. Furthermore, pain may decrease baseline levels of processing in the posterior cingulate [26].

For both evoked and spontaneous tics, activation in S1 (PoCG, Brodmann Area 2 or 3) was observed contralateral to the pain in the area corresponding to the somatotopic representation of the origin of the painful tics (i.e., face – actual pain was just lateral to the upper right lip). It was observed for both evoked and spontaneous tics (see Table 1 and Table 3). In spontaneous tics, decreased signal was observed in the same region as evoked. This decrease may reflect a refractory spontaneous response, as it was often preceded by an evoked tic.

### CNS activation by evoked tics

Prefrontal activation was observed in the superior, middle and inferior regions, and was greater contralateral to the pain. Prefrontal regions have been associated with both cognitive and modulatory components of pain [27]. The widespread activation observed in the frontal regions may also relate to the anticipation of oncoming pain [28], as well as evidence that frontal lobe circuits are more profoundly involved in chronic pain than acute pain [17]. The latter may relate to an evolving plasticity in frontal lobe function related to neural loss [29]. The observation of activation foci within the superior (dorsal), middle (lateral), and inferior frontal lobe could indicate processing in cognitive, executive, attentional and working memory including learned association [30] in response to painful events (tics).

Activation was also observed in both the posterior parietal cortex and temporal lobes (specific Brodmann Areas delineated in the Tables). Right-lateralized coincident activation of posterior parietal and prefrontal cortices may be involved in attentional and memory networks activated by noxious stimulation [31]. In this case, bilateral activation was observed with similar right sided-dominance. Temporal lobe activation is not frequently reported in pain imaging studies [18], and its role in pain processing is not known.

Perhaps somewhat surprising was the observation of almost equal activation in the right and left thalamus given the precise localization of the tic. We have observed this in imaging studies of migraine patients (Borsook et al., unpublished observations) and of patients with trigeminal neuropathy [9]. Others have observed this in non-clinical imaging studies [32]. Another surprising result was the lack of significant activation in the cerebellum for evoked tics, since most pain studies report cerebellar activation [33] as summarized in a recent meta-analysis for thermal stimuli [34]. We recently reported differences in activation in the cerebellum following trigeminal pain in patients with other trigeminal neuropathies [35].

Activation was observed in the pontine reticular formation, and has also been reported previously in healthy subjects with experimental pain [36] and in patients with neuropathic pain [9]. The area is involved in a "startle reflex", activating more rostral brain regions and spinal regions in response to threatening stimuli, and may act as an arousal system. Indeed, mesopontine reticular neurons send afferents to multiple thalamic relay nuclei that project to various cortical regions, including those observed in our study.

### Spontaneous tics

Spontaneous tics produced activation patterns that differed with evoked tics in a number of respects. First, a contrast analysis showed that evoked tics produced more activation that spontaneous tics in cortical, sub-cortical, and brainstem (pontine reticular activating system) regions (see Table 3 vs. Table 4). Second, there were differences in activation of the cerebellum, and decreased activation in the posterior cingulate (see above), amygdala, and hippocampus (see Table 4). With both spontaneous and evoked stimuli, the hypothalamus also displayed decreased activation. The exact nature of what decreased activation is not clear, but a number of authors have suggested that it represents inhibitory processing [37-41].

Many of the regions showing decreased activation have been considered as part of an integrated reward/aversion circuitry [19]. Decreased activity induced by pain has been reported in the amygdala and hypothalamus [19], as well as the posterior cingulate (see above). The severity of the pain (reported as 8/10) may be related to enhanced negative activation in this aversion circuitry. The amygdala may receive direct neural responses related to pain via the spino(trigemino)-parabrachial-amygdala tract [42]. Also, amygdalar activation may correlate with the processing of emotional reactions (e.g., anxiety and fear) to external stimuli and with the integration of defense responses [43]. The hypothalamus receives direct nociceptive inputs via the trigeminohypothalamic tract [44], and may relate to central integration of an autonomic response to pain [19].

Another region activated with spontaneous pain but not evoked pain was the hippocampus, which also showed decreased activation (Table 1). This region has been involved in a number of neural processes including pain-related anxiety [45], and comparison of actual and expected stimuli [46]. Decreased activation in the hippocampus may reflect engagement of endogenous modulatory processes (see below), or fear conditioning [47].

Differences were observed for evoked and spontaneous pain for insular single trial averages: these included differences in the onset and slope of the activation, with a smaller slope for the evoked pain and a longer onset for the BOLD response for the spontaneous pain. These differences may relate to expectation (see below) or differences in the way the subject timed her response (turning the dial) to indicate the onset of the tic.

### Expectation – evoked vs. spontaneous pain

Contrast analysis showed that evoked tics resulted in greater overall activation than for spontaneous tics. The magnitude of pain-related activations was largely greater for evoked pain than spontaneous pain. However, consistent with recent reports, subjects reported lower levels of pain intensity when the shocks were predictable [7]. Knowledge of certain and predictable pain may enhance activation patterns related to both expectation and nociceptive processing [48].

We interpret the results of the contrast analysis (Figure 6; Table 3) as brain activation related to the expectation of certain pain. As shown in the single trial average from the insula (Figure 3), the onset of the increase in the BOLD response is earlier for evoked tics than spontaneous tics, suggestive of the influence of neural systems orchestrating expectancy. Previous work in healthy subjects has reported that expectation of pain and actual encoding of noxious stimuli have overlapping representations [49]. Although expectation is typically measured preceding stimulus application, preparatory processes triggered by the threat of impending pain may alter subsequent nociceptive or other processing [50-52]. Here the experimental set-up was very clear in terms of expectant pain every 2 minutes. However, neither the patient nor the experimenters controlled the occurrence of spontaneous pain. The difference between evoked and spontaneous pain may be a result of expectancy-induced activations.

Regions predominantly activated with evoked pain, including the prefrontal cortex, anterior cingulate, hippocampus, and amygdala, are involved in the mental representation of an event [49,50,53-55]. Pathways activated during expected pain included the anterior cingulate cortex (see below), insula, and parietal cortex, and superior temporal cortex [56]; all these regions are thought to modulate expectation. Separate activations in the perigenual ACC and posterior ACC were observed for the contrast analysis of expected vs. unexpected (see Figure 6). A previous study suggests that the cingulate is functionally segregated with respect to externally generated (posterior cingulate cortex) vs. self-administered pain (perigenual ACC) [53]. Perigenual ACC activation with evoked pain in our study may correspond with its activation by self-administered pain in the Mohr study. In addition, signal in the posterior cingulate cortex (PCC) decreased with spontaneous pain (see above). Ploghaus and colleagues [54] reported that expectation of pain activated the medial frontal lobe and insula in regions that were close to but distinct from areas activated by pain. In this report, there was little temporal separation between expecting pain and the pain experience (i.e., the pain occurred immediately following the cue).

In addition, anticipation of pain likely also activates neural systems involved in the modulation of pain [57]. In the case of the tic patient, anxiety associated with triggering evoked pain may activate such systems, providing a possible route for decreased pain for evoked vs. spontaneous shocks.

### Tic vs. neuropathic pain

Activation in trigeminal neuropathy by brush, cold or heat stimuli do not produce such high levels of activation [9]. Neuropathic pain may consist of spontaneous and evoked pain. Tic doloureaux is an unusual neuropathic pain disorder in that it is usually manifest with only shooting pain, though it may also be accompanied by underlying burning pain in the area. Allodynia or hyperalgesia are normally not associated with this. Other neuropathic pains such as sciatica may also be associated with intermittent shooting pain. The shooting pain seems to be associated with similar patterns of activation as in allodynia, although the shock-like symptoms produce remarkably high activation levels.

### Caveats

There was less overall activation from spontaneous tics than evoked pain both in number of foci that were activated and the total volume of activation. We do not think that these differences were the result of head movement or tapping, as shown by Figure 2. Furthermore, proper timing of the onset and offset of each episode of pain was recorded for spontaneous pain. Thus the greater activation of evoked pain, we believe, was not simply a result of better modeling of the evoked pain.

Regions that have been correlated to motor function, such as posterior anterior cingulate cortex [58], SMA, S1, and M1 [59], may have been activated in this study in relation to the mild motor tic, and the motor planning/execution involved in the patient's marking of tic events. The bilateral motor responses may be secondary to bilateral muscle contractions that may be observed following stimulation of the nerve and that may take place in response to or in preparation for the painful tic [60,61]. This response may be generalized, but more likely to be elicited by the tic via the trigemino-facial reflex. Direct stimulation of the trigeminal nerve in patients undergoing retrogasserian thermocoagulation produces activation in the ipsilateral temporalis, masseter, and anterior belly of the digastric muscle [62] as a result of the trigeminofacial reflex. However, evaluation of motion correction parameters indicates that the position of the head was minimally affected by these motor tasks/reflexes. The patient's act of marking the occurrence of tic events perhaps also contributed to activation of M1, SMA, and posterior ACC with both evoked and spontaneous EVs.

This is an n = 1 study. The nature of the condition precludes an elegant cohort study in terms of design. Furthermore, it is very unusual to be able to control head movement in these patients in the scanner during the tics. In addition, the event related fMRI with single trial averaging may enhance the contrast to noise relative to signal [63]. In our study, 10 (evoked) and 13 (spontaneous) events were used for evaluating the brain response. Compared with previous studies of stimulus-induced pain [19], a larger number of time-points (tp) were used for the evaluation of the brain response (n ~ 404 tp used here vs. around n ~ 100 tp in previous pain studies). By using a large number of time points/number of stimuli, we decrease the variance for the observed signal. Further details of the design matrix are provided in the addendum on the web [Additional file [Supplementary-material S1]].

A potential concern for the interpretation of the data is that the evoked tic condition consisted of an array of stimuli that could potentially have different effects on the BOLD signal; namely the touch cue, the subject tapping her teeth, the subject marking pain onset, and the possibility of facial motor reflexes. However, the touch cue itself (an isolated light tap) is unlikely to produce a robust BOLD response, and the mild tapping of the teeth likely produces a marginal response that is not more than the "normal swallowing" that occurs during regular imaging of subjects [64]. The spontaneous tic condition permitted the consideration of tic-evoked activity without a cue or teeth tapping, and perhaps represents a cleaner physiological measure of tic-evoked pain. Potentially, motor systems could have been recruited during the subject's use of the dial to mark pain onset, and during possible tic-induced facial reflexes.

## Conclusion

We believe that this is the first report of brain activation in tic doloureaux. Although the syndrome may have elements seen in other neuropathic pain conditions (e.g., small areas of altered sensory function [65,66], the overwhelming feature of tic doloureaux is the severe lancinating pain that may be triggered with perturbation or may occur spontaneously. The pain is usually consistent in its nature and may vary in intensity. A number of features seem to differentiate the brain activation in tic doloureaux from evoked pain in neuropathic facial pain patients [9]. Predominant among these is the level of activation observed in the frontal regions, including the anterior cingulate cortex and the basal ganglia. Spontaneous tics observed in this study seem to activate classic aversion circuitry [19], and this may contribute to the high level of anxiety related to the disorder [23,67].

## Supplementary Material

Additional File 1 Design matrix. Demonstration that variance of the observed signal was minimized by using a large number of time points/number of stimuli. Click here for file

## References

[B1] Kumar GK Vartanian AJ Alvi A When is facial pain trigeminal neuralgia? Postgrad Med 1998 104 149 151 155-146 9793561 10.3810/pgm.1998.10.451

[B2] Truini A Galeotti F Cruccu G New insight into trigeminal neuralgia J Headache Pain 2005 6 237 239 10.1007/s10194-005-0195-9 16362674 PMC3452002

[B3] Burchiel KJ Trigeminal neuropathic pain Acta Neurochir Suppl (Wien) 1993 58 145 149 8109278 10.1007/978-3-7091-9297-9_33

[B4] Cheshire WP Jr The shocking tooth about trigeminal neuralgia N Engl J Med 2000 342 2003 10.1056/NEJM200006293422619 10877664

[B5] Dworkin RH Backonja M Rowbotham MC Allen RR Argoff CR Bennett GJ Bushnell MC Farrar JT Galer BS Haythornthwaite JA Advances in neuropathic pain: diagnosis, mechanisms, and treatment recommendations Arch Neurol 2003 60 1524 1534 10.1001/archneur.60.11.1524 14623723

[B6] Arienzo D Babiloni C Ferretti A Caulo M Del Gratta C Tartaro A Rossini PM Romani GL Somatotopy of anterior cingulate cortex (ACC) and supplementary motor area (SMA) for electric stimulation of the median and tibial nerves: an fMRI study Neuroimage 2006 33 700 705 10.1016/j.neuroimage.2006.06.030 16935009

[B7] Carlsson K Andersson J Petrovic P Petersson KM Ohman A Ingvar M Predictability modulates the affective and sensory-discriminative neural processing of pain Neuroimage 2006 32 1804 1814 10.1016/j.neuroimage.2006.05.027 16861005

[B8] Baliki MN Chialvo DR Geha PY Levy RM Harden RN Parrish TB Apkarian AV Chronic pain and the emotional brain: specific brain activity associated with spontaneous fluctuations of intensity of chronic back pain J Neurosci 2006 26 12165 12173 10.1523/JNEUROSCI.3576-06.2006 17122041 PMC4177069

[B9] Becerra L Morris S Bazes S Gostic R Sherman S Gostic J Pendse G Moulton E Scrivani S Keith D Trigeminal Neuropathic Pain Alters Responses in CNS Circuits to Mechanical (brush) and Thermal (cold and heat) Stimuli J Neurosci 2006 in press 10.1523/JNEUROSCI.2305-06.2006 PMC6674763 17050704

[B10] Maihofner C Handwerker HO Birklein F Functional imaging of allodynia in complex regional pain syndrome Neurology 2006 66 711 717 10.1212/01.wnl.0000200961.49114.39 16534108

[B11] Borsook D DaSilva AF Ploghaus A Becerra L Specific and somatotopic functional magnetic resonance imaging activation in the trigeminal ganglion by brush and noxious heat J Neurosci 2003 23 7897 7903 12944520 10.1523/JNEUROSCI.23-21-07897.2003 PMC6740587

[B12] Woolrich MW Ripley BD Brady M Smith SM Temporal autocorrelation in univariate linear modeling of FMRI data Neuroimage 2001 14 1370 1386 10.1006/nimg.2001.0931 11707093

[B13] Pendse G Borsook D Aiello-Lammens M Moulton EA Becerra L Analyzing Response Characteristics in fMRI using Logistic Regression Society for Neuroscience 2006 36

[B14] Brooks JC Zambreanu L Godinez A Craig AD Tracey I Somatotopic organisation of the human insula to painful heat studied with high resolution functional imaging Neuroimage 2005 27 201 209 10.1016/j.neuroimage.2005.03.041 15921935

[B15] Singer T The neuronal basis of empathy and fairness Novartis Found Symp 2007 278 20 30 discussion 30–40, 89–96, 216–221 17214308

[B16] Ostrowsky K Magnin M Ryvlin P Isnard J Guenot M Mauguiere F Representation of pain and somatic sensation in the human insula: a study of responses to direct electrical cortical stimulation Cereb Cortex 2002 12 376 385 10.1093/cercor/12.4.376 11884353

[B17] Apkarian AV Bushnell MC Treede RD Zubieta JK Human brain mechanisms of pain perception and regulation in health and disease Eur J Pain 2005 9 463 484 10.1016/j.ejpain.2004.11.001 15979027

[B18] Peyron R Laurent B Garcia-Larrea L Functional imaging of brain responses to pain. A review and meta-analysis (2000) Neurophysiol Clin 2000 30 263 288 10.1016/S0987-7053(00)00227-6 11126640

[B19] Becerra L Breiter HC Wise R Gonzalez RG Borsook D Reward circuitry activation by noxious thermal stimuli Neuron 2001 32 927 946 10.1016/S0896-6273(01)00533-5 11738036

[B20] Barrett AP Schifter M Trigeminal neuralgia Aust Dent J 1993 38 198 203 8373292 10.1111/j.1834-7819.1993.tb03064.x

[B21] Eskandar E Barker FG 2nd Rabinov JD Case records of the Massachusetts General Hospital. Case 21-2006. A 61-year-old man with left-sided facial pain N Engl J Med 2006 355 183 188 10.1056/NEJMcpc069011 16837683

[B22] Ruehle BS Handwerker HO Lennerz JK Ringler R Forster C Brain activation during input from mechanoinsensitive versus polymodal C-nociceptors J Neurosci 2006 26 5492 5499 10.1523/JNEUROSCI.2059-05.2006 16707801 PMC6675308

[B23] Ochsner KN Ludlow DH Knierim K Hanelin J Ramachandran T Glover GC Mackey SC Neural correlates of individual differences in pain-related fear and anxiety Pain 2006 120 69 77 10.1016/j.pain.2005.10.014 16364548 PMC2914607

[B24] Zambreanu L Wise RG Brooks JC Iannetti GD Tracey I A role for the brainstem in central sensitisation in humans. Evidence from functional magnetic resonance imaging Pain 2005 114 397 407 10.1016/j.pain.2005.01.005 15777865

[B25] Rainville P Duncan GH Price DD Carrier B Bushnell MC Pain affect encoded in human anterior cingulate but not somatosensory cortex Science 1997 277 968 971 10.1126/science.277.5328.968 9252330

[B26] Vogt BA Laureys S Posterior cingulate, precuneal and retrosplenial cortices: cytology and components of the neural network correlates of consciousness Prog Brain Res 2005 150 205 217 16186025 10.1016/S0079-6123(05)50015-3 PMC2679949

[B27] Lorenz J Minoshima S Casey KL Keeping pain out of mind: the role of the dorsolateral prefrontal cortex in pain modulation Brain 2003 126 1079 1091 10.1093/brain/awg102 12690048

[B28] Wager TD Rilling JK Smith EE Sokolik A Casey KL Davidson RJ Kosslyn SM Rose RM Cohen JD Placebo-induced changes in FMRI in the anticipation and experience of pain Science 2004 303 1162 1167 10.1126/science.1093065 14976306

[B29] Apkarian AV Sosa Y Sonty S Levy RM Harden RN Parrish TB Gitelman DR Chronic back pain is associated with decreased prefrontal and thalamic gray matter density J Neurosci 2004 24 10410 10415 10.1523/JNEUROSCI.2541-04.2004 15548656 PMC6730296

[B30] Miller EK Cohen JD An integrative theory of prefrontal cortex function Annu Rev Neurosci 2001 24 167 202 10.1146/annurev.neuro.24.1.167 11283309

[B31] Symonds LL Gordon NS Bixby JC Mande MM Right-lateralized pain processing in the human cortex: an FMRI study J Neurophysiol 2006 95 3823 3830 10.1152/jn.01162.2005 16554508

[B32] Coghill RC Sang CN Maisog JM Iadarola MJ Pain intensity processing within the human brain: a bilateral, distributed mechanism J Neurophysiol 1999 82 1934 1943 10515983 10.1152/jn.1999.82.4.1934

[B33] Helmchen C Mohr C Erdmann C Binkofski F Cerebellar neural responses related to actively and passively applied noxious thermal stimulation in human subjects: a parametric fMRI study Neurosci Lett 2004 361 237 240 10.1016/j.neulet.2003.12.017 15135937

[B34] Farrell MJ Laird AR Egan GF Brain activity associated with painfully hot stimuli applied to the upper limb: a meta-analysis Hum Brain Mapp 2005 25 129 139 10.1002/hbm.20125 15846813 PMC6871740

[B35] Borsook D Moulton E Tully S Schmahmann J Becerra L Human cerebellar responses to brush and heat stimuli in healthy and neuropathic pain subjects Cerebellum in press 10.1007/s12311-008-0011-6 18418691

[B36] Dunckley P Wise RG Fairhurst M Hobden P Aziz Q Chang L Tracey I A comparison of visceral and somatic pain processing in the human brainstem using functional magnetic resonance imaging J Neurosci 2005 25 7333 7341 10.1523/JNEUROSCI.1100-05.2005 16093383 PMC6725297

[B37] Attwell D Iadecola C The neural basis of functional brain imaging signals Trends Neurosci 2002 25 621 625 10.1016/S0166-2236(02)02264-6 12446129

[B38] Harel N Lee SP Nagaoka T Kim DS Kim SG Origin of negative blood oxygenation level-dependent fMRI signals J Cereb Blood Flow Metab 2002 22 908 917 10.1097/00004647-200208000-00002 12172376

[B39] Kobayashi E Bagshaw AP Grova C Dubeau F Gotman J Negative BOLD responses to epileptic spikes Hum Brain Mapp 2006 27 488 497 10.1002/hbm.20193 16180210 PMC6871405

[B40] Shmuel A Yacoub E Pfeuffer J Van de Moortele PF Adriany G Hu X Ugurbil K Sustained negative BOLD, blood flow and oxygen consumption response and its coupling to the positive response in the human brain Neuron 2002 36 1195 1210 10.1016/S0896-6273(02)01061-9 12495632

[B41] Stefanovic B Warnking JM Pike GB Hemodynamic and metabolic responses to neuronal inhibition Neuroimage 2004 22 771 778 10.1016/j.neuroimage.2004.01.036 15193606

[B42] Bernard JF Besson JM The spino(trigemino)pontoamygdaloid pathway: electrophysiological evidence for an involvement in pain processes J Neurophysiol 1990 63 473 490 2329357 10.1152/jn.1990.63.3.473

[B43] LeDoux J The emotional brain, fear, and the amygdala Cell Mol Neurobiol 2003 23 727 738 10.1023/A:1025048802629 14514027 PMC11530156

[B44] Malick A Burstein R Cells of origin of the trigeminohypothalamic tract in the rat J Comp Neurol 1998 400 125 144 10.1002/(SICI)1096-9861(19981012)400:1<125::AID-CNE9>3.0.CO;2-B 9762871

[B45] Ploghaus A Narain C Beckmann CF Clare S Bantick S Wise R Matthews PM Rawlins JN Tracey I Exacerbation of pain by anxiety is associated with activity in a hippocampal network J Neurosci 2001 21 9896 9903 11739597 10.1523/JNEUROSCI.21-24-09896.2001 PMC6763058

[B46] Gray JA McNaughton N The Neuropsychology of Anxiety: An Enquiry into the Functions of the Septo-Hippocampal System 2000 2 Oxford: Oxford University Press

[B47] Sanders MJ Wiltgen BJ Fanselow MS The place of the hippocampus in fear conditioning Eur J Pharmacol 2003 463 217 223 10.1016/S0014-2999(03)01283-4 12600712

[B48] Ploghaus A Becerra L Borras C Borsook D Neural circuitry underlying pain modulation: expectation, hypnosis, placebo Trends Cogn Sci 2003 7 197 200 10.1016/S1364-6613(03)00061-5 12757820

[B49] Koyama T McHaffie JG Laurienti PJ Coghill RC The subjective experience of pain: where expectations become reality Proc Natl Acad Sci USA 2005 102 12950 12955 10.1073/pnas.0408576102 16150703 PMC1200254

[B50] Keltner JR Furst A Fan C Redfern R Inglis B Fields HL Isolating the modulatory effect of expectation on pain transmission: a functional magnetic resonance imaging study J Neurosci 2006 26 4437 4443 10.1523/JNEUROSCI.4463-05.2006 16624963 PMC6674009

[B51] Helmchen C Mohr C Erdmann C Binkofski F Buchel C Neural activity related to self-versus externally generated painful stimuli reveals distinct differences in the lateral pain system in a parametric fMRI study Hum Brain Mapp 2006 27 755 765 10.1002/hbm.20217 16453310 PMC6871328

[B52] Carlsson K Petrovic P Skare S Petersson KM Ingvar M Tickling expectations: neural processing in anticipation of a sensory stimulus J Cogn Neurosci 2000 12 691 703 10.1162/089892900562318 10936920

[B53] Mohr C Binkofski F Erdmann C Buchel C Helmchen C The anterior cingulate cortex contains distinct areas dissociating external from self-administered painful stimulation: a parametric fMRI study Pain 2005 114 347 357 10.1016/j.pain.2004.12.036 15777860

[B54] Ploghaus A Tracey I Gati JS Clare S Menon RS Matthews PM Rawlins JN Dissociating pain from its anticipation in the human brain Science 1999 284 1979 1981 10.1126/science.284.5422.1979 10373114

[B55] Porro CA Baraldi P Pagnoni G Serafini M Facchin P Maieron M Nichelli P Does anticipation of pain affect cortical nociceptive systems? J Neurosci 2002 22 3206 3214 11943821 10.1523/JNEUROSCI.22-08-03206.2002 PMC6757517

[B56] Mesulam MM Mufson EJ Insula of the old world monkey. III: Efferent cortical output and comments on function J Comp Neurol 1982 212 38 52 10.1002/cne.902120104 7174907

[B57] Fields HL Pain modulation: expectation, opioid analgesia and virtual pain Prog Brain Res 2000 122 245 253 10737063 10.1016/s0079-6123(08)62143-3

[B58] Devinsky O Morrell MJ Vogt BA Contributions of anterior cingulate cortex to behaviour Brain 1995 118 Pt 1 279 306 10.1093/brain/118.1.279 7895011

[B59] Mima T Sadato N Yazawa S Hanakawa T Fukuyama H Yonekura Y Shibasaki H Brain structures related to active and passive finger movements in man Brain 1999 122 Pt 10 1989 1997 10.1093/brain/122.10.1989 10506099

[B60] Cruccu G Bowsher D Intracranial stimulation of the trigeminal nerve in man. II. Reflex responses J Neurol Neurosurg Psychiatry 1986 49 419 427 3701351 10.1136/jnnp.49.4.419 PMC1028769

[B61] Ongerboer de Visser BW Goor C Electromyographic and reflex study in idiopathic and symptomatic trigeminal neuralgias: latency of the jaw and blink reflexes J Neurol Neurosurg Psychiatry 1974 37 1225 1230 4457616 10.1136/jnnp.37.11.1225 PMC494886

[B62] Cruccu G Intracranial stimulation of the trigeminal nerve in man. I. Direct motor responses J Neurol Neurosurg Psychiatry 1986 49 411 418 3701350 10.1136/jnnp.49.4.411 PMC1028768

[B63] Liu HL Huang JC Wang JJ Wan YL Wai YY The effects of single-trial averaging on the temporal resolution of functional MRI Magn Reson Imaging 2006 24 597 602 10.1016/j.mri.2005.12.016 16735181

[B64] Martin R Barr A Macintosh B Smith R Stevens T Taves D Gati J Menon R Hachinski V Cerebral cortical processing of swallowing in older adults Experimental brain research Experimentelle Hirnforschung 2007 176 12 22 10.1007/s00221-006-0592-6 16896984

[B65] Bowsher D Miles JB Haggett CE Eldridge PR Trigeminal neuralgia: a quantitative sensory perception threshold study in patients who had not undergone previous invasive procedures J Neurosurg 1997 86 190 192 9010417 10.3171/jns.1997.86.2.0190

[B66] Sinay VJ Bonamico LH Dubrovsky A Subclinical sensory abnormalities in trigeminal neuralgia Cephalalgia 2003 23 541 544 10.1046/j.1468-2982.2003.00581.x 12950380

[B67] Zvolensky MJ Goodie JL McNeil DW Sperry JA Sorrell JT Anxiety sensitivity in the prediction of pain-related fear and anxiety in a heterogeneous chronic pain population Behav Res Ther 2001 39 683 696 10.1016/S0005-7967(00)00049-8 11400712

[B68] FMRIB Software Library http://www.fmrib.ox.ac.uk/fsl/

