# Supplementary figures and images for: Comparison of evoked vs. spontaneous tics in a patient with trigeminal neuralgia (tic doloureux)
Source: Mol Pain. 2007 Nov 6;3:34. doi: 10.1186/1744-8069-3-34 (PMC2217520; doi:10.1186/1744-8069-3-34)

**
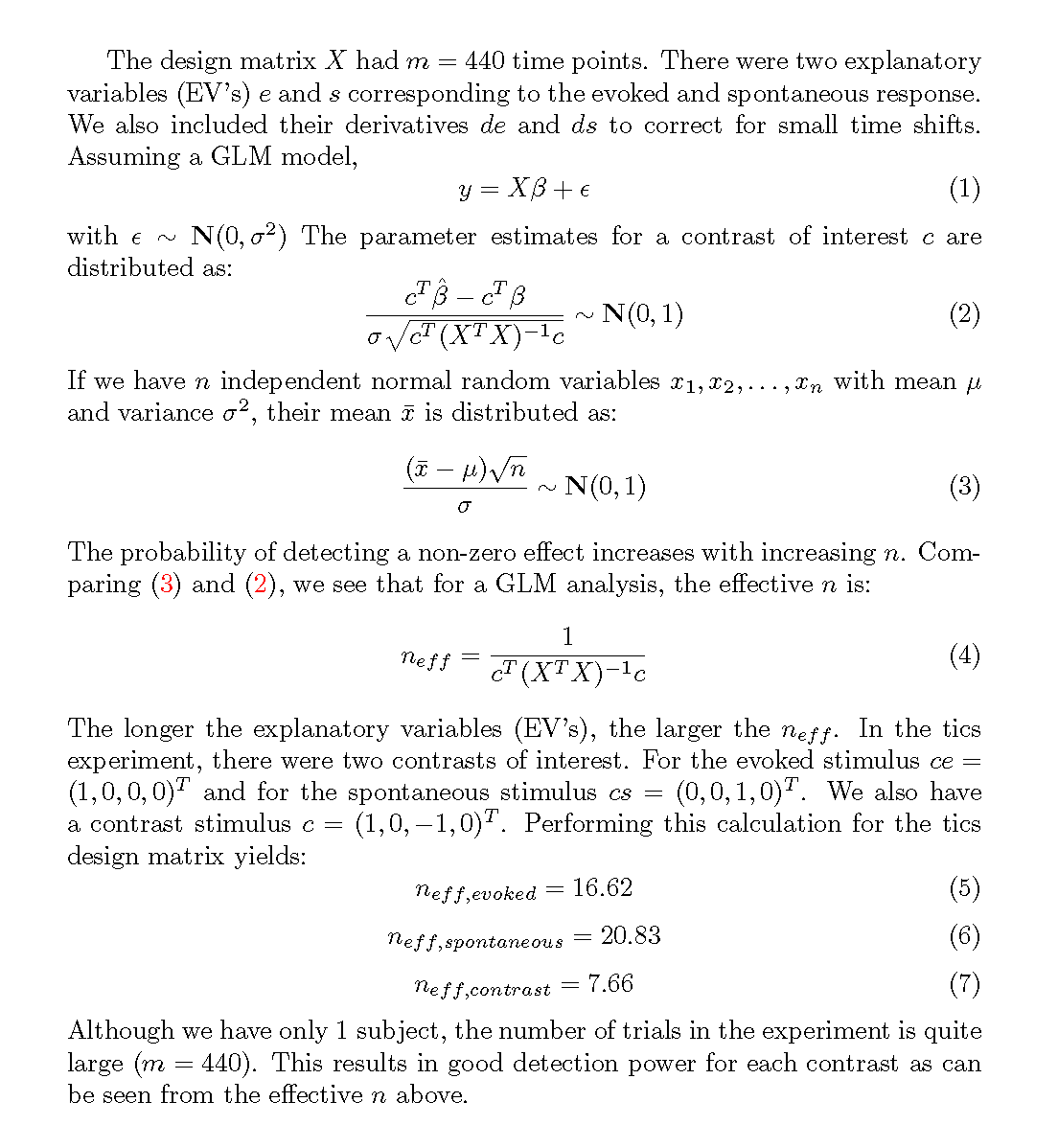
**

Supplement: Additional File 1 — Design matrix. Demonstration that variance of the observed signal was minimized by using a large number of time points/number of stimuli. [file 1744-8069-3-34-S1.doc]
